# Analyzing Cervical Microbiome Composition in HIV-Infected Women with Different HPV Infection Profiles: A Pilot Study in Thailand

**DOI:** 10.3390/microorganisms12071298

**Published:** 2024-06-26

**Authors:** Kanya Preechasuth, Lionel Brazier, Woottichai Khamduang, Sayamon Hongjaisee, Nantawan Wangsaeng, Nicole Ngo-Giang-Huong

**Affiliations:** 1 Division of Clinical Microbiology, Faculty of Associated Medical Sciences, Chiang Mai University, Chiang Mai 50200, Thailand; woottichai.k@cmu.ac.th; 2 Maladies Infectieuses et Vecteurs: Écologie, Génétique, Évolution et Contrôle (MIVEGEC), Centre National de la Recherche Scientifique (CNRS UMR5290), Institut de Recherche Pour le Développement (IRD224), Université of Montpellier, 34394 Montpellier CEDEX 5, France; lionel.brazier@ird.fr (L.B.); nicole.ngo-giang-huong2@ird.fr (N.N.-G.-H.); 3 LUCENT International Collaboration, Faculty of Associated Medical Sciences, Chiang Mai University, Chiang Mai 50200, Thailand; sayamon.ho@cmu.ac.th; 4 Research Institute for Health Sciences, Chiang Mai University, Chiang Mai 50200, Thailand; 5 AMS-PHPT Research Collaboration, Faculty of Associated Medical Sciences, Chiang Mai University, Chiang Mai 50200, Thailand; nantawan.wangsaeng@phpt.org

**Keywords:** vaginal microbiome, human papillomavirus, NGS, community state types

## Abstract

We conducted a pilot study to analyze the microbiome in cervical samples of women living with HIV with various profiles of HPV infections. The participants had an average age of 41.5 years. Sequence analysis of 16S rRNA V3 gene amplicons was performed using next-generation sequencing technology (Ion Torrent PGM^TM^). The bioinformatics pipeline was analyzed using the Find, Rapidly, OTUs with Galaxy Solution system (FROGS). Common genera were determined to identify Community State Types (CSTs). The cervical microbiome profiles showed a dominance of lactobacilli in 56% (five out of nine) of samples. All three women with normal cervical cells and high-risk HPV infection were classified as CST IV, characterized by anaerobic bacteria associated with bacterial vaginitis, such as *Gardnerella*, *Prevotella*, *Atopobium*, and *Sneathia*. Among the two women with abnormal cervical cells and high-risk HPV infection, one was classified as CST III, and the other had an unclassified profile dominated by *L. helveticus*. Four women with normal cervical cells and no HPV infection exhibited various CSTs. Our study demonstrated the feasibility of the protocol in analyzing the cervical microbiome. However, further analysis with a larger number of longitudinal samples is necessary to determine the role of cervical microbiota in HPV persistence, clearance, or the development of precancerous lesions.

## 1. Introduction

The cervicovaginal microbiome composition is thought to play a major role in the genital tract health of women [1,2]. The cervicovaginal microbiome comprises numerous bacterial species, of which lactobacilli are the most abundant species. Next-generation sequencing (NGS) approaches have been commonly used since they allow for the in-depth analysis of bacterial 16S ribosomal RNA (rRNA) and provide a detailed microbial community structure. Based on the relative abundance of rRNA, cervical bacterial communities have been categorized into five groups named Community State Types (CSTs). The dominant bacteria species in CST I, II, III, and V are *Lactobacillus crispatus*, *L. gasseri*, *L. iners*, and *L. jensenii*, respectively, while CST IV has a larger diversity and includes anaerobic bacteria such as members of genus *Atopobium*, *Prevotella*, *Sneathia*, or *Gardnerella* [3]. 

Cervical cancer is the fourth leading cause of cancer-related deaths in women worldwide, with most of the cancers caused by HPV [4]. As a result of the advances in sequencing technologies, numerous research studies have assessed the relationships between the cervical microbiome and infection/persistence/clearance of human papillomaviruses (HPV) in women’s cervix. A growing body of evidence suggests that the increased diversity of vaginal microbiota combined with a reduction in the relative abundance of lactobacilli may be involved in HPV acquisition, severity of cervical intra-epithelia neoplasia (CIN) and the development of cervical cancer [1,2]. However, the cervical microbiome composition can vary with ethnicity [3,5] and host immunity [6], which in turn may impact HPV infection and persistence. Thus, analysis of the cervical microbiome should consider these factors.

Women living with HIV (WLWH) are at higher risk of having high-risk (HR)-HPV infection and an abnormal Pap smear compared to HIV-uninfected women [7,8,9]. Furthermore, it has been reported that the microbiomes of women living with HIV and co-infected with HPV have a higher frequency to be categorized into CST III and CST IV [10]. WLWH had higher bacterial richness than HIV-negative women. *Mycoplasmatales*, *Pseudomonadales*, and *Staphylococcus* were the most abundant operational taxonomic units (OTUs) associated with high-grade squamous intraepithelial lesions (HSIL) and it suggested that inflammation due to chronic cervical mycoplasma infection may contribute to HPV-dependent dysplasia [11]. 

Data on the cervical microbiome of 25 women in Thailand indicate that two microbiome communities are frequently found predominant of lactobacilli, with *L. iners* being the most common species and non-lactobacilli-dominated with *Gardnerella* and *Atopobium* the most frequently found [12,13]. However, there is still a lack of extensive profiles of the cervical microbiome composition among HIV-positive women with different cervical cancer screening results (including Pap smear outcomes and HPV genotypes) in Thailand. Further studies are required to understand the role of cervical microbiota in HPV persistence, clearance, and the development of precancerous lesions.

In this pilot study, we explored the microbiome communities in WLWH using NGS technology with varying cervical cancer screening results, including Pap smear and HPV genotypes. We demonstrated the feasibility of using NGS technology to longitudinally investigate the role of cervical microbiota in HPV persistence, clearance, and the development of precancerous lesions. 

## 2. Materials and Methods

### 2.1. Subjects and Sample Collection

Cervical samples used in this study were collected from ten women enrolled in the Papillo V study [14] and kept stored at −70 °C until use. The samples were randomly selected based on the results of Pap smear and HPV testing. In the “Normal” group, cervical swabs were collected from women with normal Pap smear (Normal) and no presence of HPV (Negative); Normal/Negative (NN). A second group included samples from women with high-grade squamous intraepithelial lesions (HSIL) on Pap smear and infection with high-risk (HR) HPV; HSIL/HR-HPV (HH). The third group included samples from women with normal Pap smear and infection with HR-HPV; Normal/HR-HPV (NH).

### 2.2. DNA Extraction

Bacterial genomic DNA was extracted using the NucleoSpin^®^Tissue (Macherey-Nagel, Düren, Germany) according to the manufacturer’s instructions. The quality and quantity of DNA was measured by the Nanodrop as preliminary data. The DNA was stored at −70 °C until further processing.

### 2.3. DNA Libraries Preparation

A fragment of 181 bp of the V3 region of the 16S rRNA gene was amplified using primers Probio_Uni and Probio_Rev that included a 10-mer barcode and adaptor sequences needed for the Ion Torrent PGMTM sequencing technology. The libraries were prepared using the fusion method as previously reported with slight modifications [15,16]. Briefly, each DNA extract was amplified in four PCR reactions to avoid bias. For each PCR reaction, one microliter of DNA extract was added to 4 µL of 5× High-Fidelity PCR master mix, (Thermo Fisher, Paris, France), 0.4 µL of 10 µM Probio_Uni primer, 0.4 µL of 10 µM Probio_Rev primer, 0.4 µL of 5 mM dNTP, and 0.4 units Q5 DNA polymerase (New England Biolabs, Ipswich, MA, USA) in a final volume of 20 µL. PCR conditions were as follows: denaturation at 98 °C for 30 s, followed by 30 cycles at 98 °C for 30 s, at 50 °C for 30 s, at 72 °C 15 s, and final extension at 72 °C for 10 min. The four PCR products were pooled and purified twice using the AGENCOURT AMPure XP reagent (Beckman Coulter, Inc., Atlanta, GA, USA), then quantified with the Agilent 2100 bioanalyzer and high-sensitivity DNA kit (Aligent Technologies, Wald Bronn, Germany).

### 2.4. 16S RNA Gene Sequencing

The purified DNA libraries were pooled in equal concentrations (100 pM) then underwent emulsion PCR with the Ion PGM^TM^ Hi-Q^TM^ OT2 Kit in the Ion OneTouch^TM^ 2 System (Life Technologies, Paris, France) according to the manufacturer ’s instructions. The bar-coded amplicon libraries were sequenced using the Ion PGM™ Hi-Q™ sequencing kit after loading on an Ion 318™ chips (Life Technologies, Paris, France). The raw sequences were pre-processed by deleting the adapter, then data files were converted to the Fastq format by the Ion Torrent Suite^TM^ Software version 5.12.0 (Life Technologies, Paris, France). Acceptable sequence reads from Ion Torrent platform include chip loading efficiency above 60–70%, the number of reads meeting the expected output for the specific chip type are 5 million read for Ion 318 chips and reads with quality scores ≥ Q20.

### 2.5. Sequence Analysis

The Fastq data files were analyzed using the bioinformatics pipeline of Fast, Rapidly, OTUs with Galaxy Solution (FROGS) 3.2 [17]. Shortly, the raw sequences from Fastq files were pre-processed (FROGS pre-process) for deletion of sequences with unexpected lengths or with ambiguous bases, merging of reads, and dereplication. Sequences obtained were then clustered (FROGS clustering swarm) and PCR chimera in each sample were removed (FROGS remove chimera). Operational Taxonomic Unit (OTU) abundance was analyzed and OTUs with more than 10 were kept (FROGS OTU filters). Taxonomic identification of OTUs was performed using Ribosomal database project (RDP) classifier and the Silva138.1 16S database (FROGS Affiliation OTU). Finally, taxonomy distribution within each sample was obtained (FROGS affiliation stat). Genus-level identification was accepted at more than 97% sequencing identity. The person performing NGS and OTU classification was blinded to the women and samples’ characteristics.

## 3. Results

### 3.1. Characteristics of Women and Samples

Cervical samples were collected from ten HIV-infected women on antiretroviral therapy [14]. Their characteristics and their Pap smear and HPV genotyping results are described in Table 1. Their mean age was 41.5 years, ranging from 31 to 61 years, and their mean CD4 count was 600 cells/mm^3^, ranging from 370 to 1253 cells/mm^3^. 

### 3.2. Analysis of Cervical Microbiome

**Figure 1:**
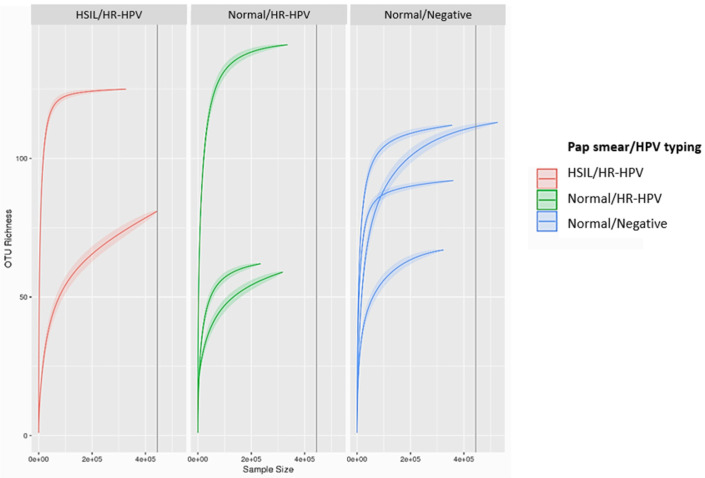
Rarefaction curves calculated with OTUs richness of HH1 and HH2 (red), NH1-NH3 (green), and NN1-NN4 (blue).

Amplification and sequencing were successfully performed on all samples. The summary of the run showed a loading density of 61%, usable reads at 71% with a total of 4,804,855 reads, and a median read length of 189 bp. Each sample contained an average of 448,695 reads (SD 97,128 reads) and sufficient OTU richness to represent species identity, shown in rarefaction curves reaching a plateau (Figure 1). The negative control had 1998 reads. One sample, HH 3 was excluded in the further analysis since the number obtained after OTU affiliation was 15,401, which could have occurred from contamination with exogeneous DNA.

### 3.3. Composition of Bacterial and CST Typing

**Figure 2:**
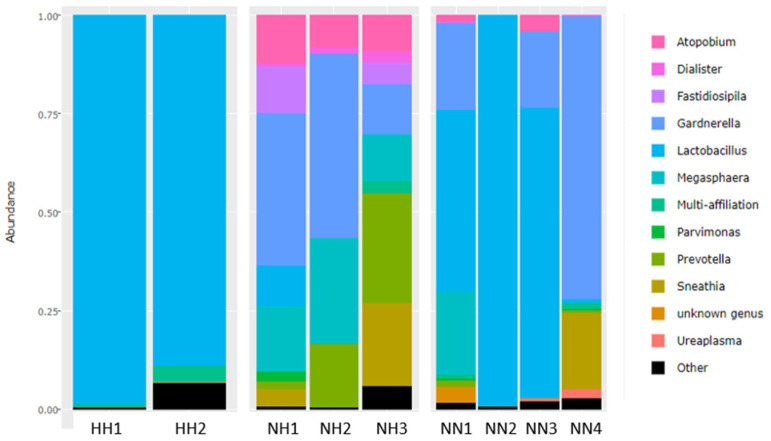
The proportion of the twelve most common genera in each sample.

Raw FASTAQ file data were analyzed with FROGS [17] and showed that most common genus (*Lactobacillus*, *Gardnerella*, *Megashaera*, and *Atopobium*) were present in the vaginal microbiome. The relative abundance of the twelve most frequent genera in each sample is shown in Figure 2. Three samples (HH1, NN2, NN3) belonged to CSTIII characterized by the predominance of *L. iners* (73–95%). Four samples (NH1, NH2, NH3, NN4) belonged to CST IV, characterized by diverse bacterial microbiota but predominantly of *G. vaginalis* (39–47%), Megasphaera, and Prevotella. One sample (NN1) was classified as CST III/IV as almost half was *L. iners* and it was diverse of anaerobic bacterial microbiota. One sample (HH2) including mostly *L. helviticus* was unclassified CST, and is shown in Table 2.

## 4. Discussion

Various regions of 16S rRNA can be used as sequencing targets, such as V3, V3-V4, V4 and V6-V7 regions [18,19]. Our study focused on the use of the V3 region of 16S rRNA to analyze the cervical microbiome, employing the Probio_Uni and Probio_Rev primers (181 bp) optimized for the Ion Torrent PGM^TM^ system [15]. Despite the small size of the amplicons, our results successfully identified key bacterial phyla (Firmicutes, Actinobacteria, Bacteroidetes and Fusobacteria), as well as common genera and species. This demonstrates that the V3 region is effective for identifying CSTs in cervical microbiome studies. Our finding aligns with the study by Sirichoat et al., which indicated that the V3 region (approximately 215 bp) yielded the highest diversity of bacterial species compared to the V6–V7, V4, and V9 regions [19]. 

Our study was exploratory with a few samples in each group, limiting the depth of our conclusions. We observed that four women had CST III, which is characterized by the predominance of *L. iners* that has been associated with a higher risk of bacterial and viral infections and the development of precancerous lesions in Caucasian women [1,20]. However, *L. iners* is commonly found in Asian women and may not indicate a similar risk of viral infection or precancerous lesions in that context. In our study, only one of three women with the predominance of *L. iners* had a high-risk HPV infection.

We also found *L. helveticus* in one sample from a woman with high-risk HPV infection and altered cervical cells (HH2). This species was reported as one of vaginal microbiota in Indian women who have bacterial vaginitis [20,21]. Additionally, *L. helveticus* has been suggested to improve the vaginal health index in perimenopausal women with bacterial vaginosis, indicating a positive role in maintaining a healthy vaginal microbiome [22]. *L. crispatus* and *L. gasseri* which are common in White or European women, were absent in our study, consistent with their lesser prevalence in Asian, Black and Hispanic women [3]. The absence of these species suggests a potentially reduced ability to clear HPV in the population of Chinese women [23].

This pilot study aimed to assess the microbiome community in cervical samples of women living with HIV on antiretroviral therapy (ART). A previous meta-analysis reported that ART was associated with a lower prevalence of high-risk HPV compared to those not on ART [24]. Additionally, ART did not appear to disrupt microbial communities, as evidenced by the study by Liu et al., which showed that the vaginal microbiome remained stable post-ART initiation in women from sub-Sahara Africa [25]. Therefore, the microbiome results in our study are likely not affected by ART.

## 5. Conclusions

The protocol employed in this pilot study demonstrated the feasibility of identifying CSTs in most of the samples. However, due to the limited number of samples evaluated, no clear conclusion could be drawn regarding the relationship between CSTs and the risk of HPV infection or lesions. Future studies with larger and longitudinal sample sets are necessary to understand the role of cervical microbiota in HPV infection and persistence. This knowledge could potentially lead to treatments or prevention strategies for cervical precancerous lesions through the replacement with beneficial cervical microbiota.

## Figures and Tables

**Table 1:** Samples’ characteristics (N = 10).

Results of Pap Smear/HPV Typing *	Sample Code	Ages	Level of CD4 (cells/mm^3^)	HPV Type
HSIL/HR-HPV	HH 1	33	408	16
	HH 2	42	637	52
	HH 3	61	508	52
Normal/HR-HPV	NH 1	31	624	16, 33, 39, 58, 40
	NH 2	40	537	52
	NH 3	42	307	52
Normal/Negative	NN 1	31	464	Negative
	NN 2	35	1253	Negative
	NN 3	40	600	Negative
	NN 4	60	601	Negative

* HSIL: high-grade squamous intraepithelial lesions, HR-HPV: high-risk HPV, Normal: normal Pap smear result, Negative: no presence of HPV.

**Table 2:** Predominant microbiome in cervical samples by taxonomic distribution.

Bacterial Microbiota	Percentage of Bacteria Microbiota in Samples								
	HH1	HH2	NH1	NH2	NH3	NN1	NN2	NN3	NN4
*Lactobacillus iners*	73.46		10.16			46.13	95.39	73.42	
*L. helveticus*	25.40	89.15					3.66		
*Gardnerella vaginalis*			38.73	46.94	12.87	21.98		19.46	71.68
*Megasphaera*			15.75	26.56	11.79	20.99			
*Sneathia*			4.49		21.02				19.29
*Atopobium vaginae*			12.49	8.37	9.30	1.72		4.25	
*Prevotella*				16.02	28.03				
*Fastidiosipila*			11.88		2.57				
*Ureaplasma*		0.14							2.32
Community State Type (CST)	III	Unclassified	IV	IV	IV	III/IV	III	III	IV

## Data Availability

The original contributions presented in the study are included in the article, further inquiries can be directed to the corresponding author.
